# Characterization and optimization of polymer-polymer aqueous two-phase systems for the isolation and purification of CaCo2 cell-derived exosomes

**DOI:** 10.1371/journal.pone.0273243

**Published:** 2022-09-02

**Authors:** Abril Torres-Bautista, Mario A. Torres-Acosta, José González-Valdez

**Affiliations:** 1 School of Engineering and Science, Tecnológico de Monterrey, Monterrey, Nuevo León, Mexico; 2 Department of Biochemical Engineering, The Advanced Centre for Biochemical Engineering, University College London, London, England, United Kingdom; Tribhuvan University, NEPAL

## Abstract

Exosomes are cell-derived vesicles that present attractive characteristics such as nano size and unique structure for their use as drug delivery systems for drug therapy, biomarkers for prognostic, diagnostic and personalized treatments. So far, one of the major challenges for therapeutic applications of exosomes is the development of optimized isolation methods. In this context, aqueous two-phase systems (ATPS) have been used as an alternative method to isolate biological molecules and particles with promising expectations for exosomes. In this work, fractionation of exosomes obtained from CaCo2 cell line and culture media contaminants were individually performed in 20 polymer-polymer ATPS. The effect of design parameters such as polymer composition, molecular weight, and tie-line length (TLL) on polyethylene glycol (PEG)-Dextran, Dextran-Ficoll and PEG-Ficoll systems was studied. After partition analysis, 4 of the 20 systems presented the best exosome fractionation from contaminants under initial conditions, which were optimized via salt addition (NaCl) to a final concentration of 25 mM, to improve collection efficiency. The PEG 10,000 gmol-^1^ –Dextran 10,000 gmol-^1^ system at TLL 25% w/w with NaCl, showed the best potential isolation efficiency. Following this proposed strategy, an exosome purification factor of 2 in the top PEG-rich phase can be expected furtherly demonstrating that ATPS have the potential for the selective recovery of these promising nanovesicles.

## Introduction

Exosomes are biological and endogenous nano-sized (30–150 nm) membrane vesicles [1, 2] produced by normal and pathological cells which can be obtained from virtually all body fluids [3]. Exosomes are originated from late endosomes and consist of a lipid bilayer membrane [1, 2] which encapsulates cytosol and different molecular components such as associated proteins, genetic materials (i.e., DNA, mRNA, miRNA), enzymes, receptors, and their own unique biomarkers, like CD63, CD9 and CD81 [4–6] which are also present in their origin cell. These naturally occurring nanoparticles are released from the cell upon fusion of a multivesicular body (MVB), containing exosomes produced in late endosome formation, with the plasma membrane. Once they are secreted to the extracellular environment, exosomes play an important role in cell communication by transferring and facilitating the exchange of their bioactive molecules contained into a specific recipient cell based on their composition [2, 7]. To date, exosomes have demonstrated to play a crucial role in maintaining cellular homeostasis and as mediators in anti-tumor responses [3, 8]. With this context, exosomes have appeared as a very powerful and promising alternative to harness the power of intercellular communication in multiple therapeutic applications.

Due to their natural characteristics such as size and unique structure, exosomes can overcome some molecule transport limitations and have also shown multiple advantages over synthetic nanoparticles in pharmaceutical applications [2] offering a high cargo capacity, longer circulating half-life, well body toleration, excellent cellular internalization, no cytotoxic effects, and target specificity [4–6]. For those reasons, they are being explored as nanodevices for the development of new therapeutic and diagnostic tools, such as biomarkers for prognostic and diagnostic of disease, nanocarriers for drug therapy and personalized treatments [9, 10].

So far, the major challenges for exosomal therapeutic applications are the production of heterogeneous and productive exosomes, and the development of optimized methods for their isolation and storage in specific formulations [11]. To date, there are several traditional and novel methodologies for exosome isolation from complex samples like human fluids, with their corresponding advantages and disadvantages for clinical trials, point-of-care settings, and scale-up scenarios [10]. Traditional methods include centrifugation/ultracentrifugation, filtration, microfluidic isolation, polymeric precipitation isolation, size-exclusion, and liquid chromatography techniques [1, 9, 10, 12, 13]. However, these methods present several common limitations such as large equipment requirements, expensive reagents, complex and costly operating procedures, and low productivity, purity, and yields [10, 11]. To date, ultracentrifugation is the most used methodology in exosome procurement, but novel strategies are emerging such as immunoaffinity, microdevices, liquid-liquid extraction and hydrophobic interaction chromatography with promising results for yield, purity, and purification factor; while offering advantageous features such as scalability, lower processing times, effectiveness, lower economical burdens and in some cases, portability [8, 12–15]. In summary, there is a strong need to standardize effective, fast, suitable, accurate and reliable methods for exosome separation that achieve high product concentrations and purity levels, ensuring nanoparticles free of polluting materials [10]. This situation has raised the need to seek alternative methods for exosome purification and isolation. In this context, aqueous two-phase systems (ATPS) are believed to present advantages over other methodologies.

ATPS is a liquid-liquid extraction technique in which two liquid solutions (typically a polymer and a salt or two different polymer solutions) are mixed above a critical concentration to form two immiscible phases composed mainly of water [16, 17]. When a particular mixture is added to the systems, the physicochemical interactions between the molecules in the sample mixture, the phase forming chemicals along with different system design parameters offer an environment in which molecules partition to either the top or bottom phase [18]. If conditions are ideal, and the molecules of interest migrate to a phase different from that of the contaminants, a primary purification of said molecules can be achieved. Because ATPS are mainly composed of water, this purification strategy presents interesting advantages for the recovery and purification of exosomes. Among them we can mention biological compatibility, low interfacial tension, high load capacity and scale up easiness [19]. Additionally, ATPS are considered easy to operate, fast, economically feasible and non-destructive, maintaining the biological activity and specific functions of the sample components [16, 19–21]. After ATPS operations, several purification steps need to be added but the number of procedures is still reduced when ATPS are used as a primary recovery strategy. This also provides different advantages such as a reduced amount of contaminant residues which also become easier to remove [19, 22]. With this, ATPS-recovered exosomes would continue to present good biological integrity and performance [19]. Their back extraction would then consist in the removal of the ATPS phase-forming chemicals which can be achieved by different strategies including ultrafiltration/diafiltration or precipitation [22].

Several publications involving protein fractionation in polymer–polymer ATPS have been published [9, 18]. Some works have aimed to evaluate the influence of intrinsic parameters on the partition behavior of two biomolecules. Among the most important design parameters, the partition coefficient (K_P_), volume ratio (V_R_) and tie-line length (TLL) have been reported [16]. K_P_ is defined as the ratio of the particle concentration between the top and bottom phase [9, 23]. Volume ratio (V_R_) is established as the volume relation of the top and the bottom phases [18]. Tie-line length (TLL) on its part, is related to the relationship between phase-forming chemical mass composition in each of the system phases and is represented in % w/w units. The end points of a tie-line determine phase compositions at equilibrium and lie in the binodal curve, which indicates the separation between the two immiscible phases [16].

As mentioned, in ATPS molecules tend to partition to either the top or bottom phase, but the differences in phase densities and interfacial tension can be affected by factors such as high polymer molecular weights and TLLs which might cause larger particles such as cells, organelles and exosomes to be retained in the interphase [9, 24]. Since this represents a quantification challenge the interphase is usually considered as part of the bottom phase as it is the case in the present study and will be described later.

Recently, ATPS have been explored as a primary recovery strategy to isolate and purify exosomes from different sources [9, 25–28]. Examples of exosome extraction from plasma have been explored by Slyusarenko (2021) *et al*.; from urine by Shin (2018) *et al*.; from plant, cell culture, and parasite culture sources by Kırbaş (2019) *et al*.; and from tumor interstitial fluids by Shin (2015) *et al*. and Kim (2015) *et al*.

In this work, CaCo2 a colon cancer cell line was chosen as exosome source from cell culture samples, instead of human samples. This cell line is well-described and has been thoroughly used as an *in vitro* model in exosome production with higher recovery results when compared to other available mammalian cell lines [23]. CaCo2 cells have also been used for exosome procurement because of their availability and scale-up easiness, and their ability to generate heterogeneous and productive exosomes with similar quality [1, 23, 28]. Exosomes produced by these cells present typically particle diameters between 30 and 200 nm [13, 29].

The objective of the present work was to understand the effect of system design parameters (i.e., polymer composition, molecular weight, and TLL) on the discrete partition behavior of exosomes obtained from a CaCo2 cell line and the contaminants present in their culture media on 20 different polymer-polymer ATPS. This was done to propose a primary recovery strategy for these nanoparticles applicable to traditional cell culture conditions. The systems that presented the best performance were selected for further optimization by the addition of sodium chloride (NaCl) to improve the efficiency of collection. A potential ATPS strategy for the isolation and purification of exosomes from culture media contaminants is proposed with the aim of implementing it in intensified strategies for exosome procurement. Additionally, a minor economic modelling was performed to calculate the current production cost at a laboratory scale. This model was used to test a range of variables that directly impact production costs. Lastly, results were contrasted with a commercially available recovery kit for exosomes to determine scenarios where the process developed here can economically compete with a real-life product.

## Materials and methods

### Cell culture

Human colon cancer cell line CaCo2 (ATCC®, HTB37™) was cultured with DMEM-F12 medium supplemented with 10% exosome-depleted Fetal Bovine Serum (FBS; Gibco, USA) and 1% Penicillin-Streptomycin solution (Gibco, USA) at 37°C and 5% CO_2_ atmosphere. CaCo2 cells (~1x10^6^ cells/mL) were placed in 7 mL of media in 100mm x 20mm tissue-culture treated petri dishes (Corning, NY) for 48 h until cultures reached ~80% confluence. Then, the exosome-enriched culture medium was recovered and reserved in -20°C until use. Another 7 mL of fresh culture media were added to the same dishes and cells were cultured for an additional 24 h at the same conditions. The maintenance medium was also recollected and reserved. Both reserved culture media were used to isolate exosomes secreted by the cells as it is described in the next section.

### Exosome isolation

The reserved exosome-enriched culture medium was centrifuged at 300 *x g* for 10 minutes at 4°C to remove cells and debris. The supernatant was carefully collected and transferred to a new tube and centrifuged at 2000 *x g* for 5 minutes at 4°C. The obtained supernatant was then filtered using a 20 μm surfactant-free cellulose acetate (SFCA) filter (Corning, USA) and later ultracentrifuged at 125,000 *x g* for 90 minutes and 4°C using a SW 32 Ti rotor (Beckman Coulter, CA) to isolate exosomes. Finally, the supernatant was decanted without disturbing the pellet and preserved at -20°C until use, while the exosome pellet was suspended in 200 μL of 10 mM pre-filtered phosphate buffered saline (PBS; Caisson Labs, USA) at pH 7.2. Exosomes were concentrated using Amicon® Ultra-4 Centrifugal Filters Ultracel® -10K NMWL (Merck Millipore, Ireland) and stored at -80°C until use. Exosomal protein concentration was determined by measuring absorbance at 280 nm in a NanoDrop® 1000 Spectrophotometer (Thermo Scientific, USA), using Bovine Serum Albumin as standard for the corresponding calibration curve, as described in section 2.2.5.

### Exosome characterization

Exosomes were characterized by size distribution, zeta potential and the presence of exosomal marker CD63. Size distribution and zeta potential were performed by dynamic light scattering (DLS) using a Zetasizer Nano ZSP equipment (Malvern Instruments, Worcestershire, UK) at room temperature (~25°C), 175 degrees angle and 633-nm laser [4]. The sample was prepared in a standard Spectrophotometer cuvette (10 mm path length) considering the addition of 10 μL of sample diluted in 900 μL of bi-distilled water to measure particle size. The same diluted sample was used to measure zeta potential using a folded capillary zeta cell. Exosomal marker CD63 was detected in the samples by ExoELISA-Ultra CD63 Kit (System Biosciences, USA) following manufacturer instructions. Briefly, the sample was incubated at 37°C for 1 h on the provided micro-titer plate. After incubation and wash step, CD63 primary antibody was added to each well and incubated for 1 hour. After washing, the same incubation conditions were used for the secondary antibody. Then, super-sensitive TMB ELISA substrate was added and incubated at room temperature for 15 mins with shaking. After the addition of the stop buffer, the presence of CD63 was visually verified.

### Construction of polymer-polymer ATPS

ATPS construction was followed as established by González-Valdez (2011) *et al*., selected PEG-Ficoll, PEG-Dextran and Ficoll-Dextran systems gave place to 20 systems identified in Table 1 and were chosen to understand the effect of system composition, polymer MW and TLL on the partition behavior of exosomes and contaminants (i.e., the supernatant preserved after ultracentrifugation representing the cell culture media) in a discrete way. Polymer stock solutions were prepared with predetermined quantities of each polymer: PEG of nominal molecular weight of 3,350 (PEG 3,350; 50%w/w) and 10,000 (PEG 10,000; 40%w/w) gmol^−^^1^; Dextran of 10,000 (Dextran 10; 40%w/w), 70,000 (Dextran 70; 40%w/w) and 110,000 (Dextran 110; 30%w/w) gmol^−^^1^; and Ficoll of 400,000 (Ficoll 400; 40%w/w) gmol^−^^1^. The composition of each system was designed using the binodal curves presented by Zaslavsky (1994) for systems 1 to 16 [30] and by Croll *et al*. (2003) for systems 17 to 20 [31]. Each system was prepared in 1.7 mL centrifugation tubes with a final mass of 0.5 g considering the addition of 10% w/w of the sample solution, the estimated amount of each polymer stock solution according to their respective final polymer composition (%w/w), and bi-distilled water as needed. In each system 50 μg of concentrated exosomes diluted in 50 μg of 10 mM PBS or 50 μg of recovered supernatant media with around 350 μg of protein contaminants were added separately as samples. Then, the constructed systems were vortexed and dispersed by mixing for 15 min. To speed-up phase separation and equilibrium, the ATPS tubes were centrifuged at 10,000 *x g* for 10 min at 4°C using an Eppendorf centrifuge 5804 R (Eppendorf, Germany). This centrifugation step is only used to accelerate phase formation and exosome separation, which can be also achieved by letting the ATPS settle at room temperature, however, under these conditions diffusion occurs very slowly [32]. For discrete experiments, V_R_ and pH were kept constant at 1.0 and 7.0, respectively, for all systems. All experiments were carried out at least three times.

**Table 1:** Composition and parameters of selected polymer-polymer ATPS for the fractionation of exosomes and their contaminants.

System	Polymer 1 MW (g mol^-1^)	Polymer 2 MW (g mol^-1^)	TLL	Polymer 1 (%w/w)	Polymer 2 (%w/w)
1	PEG 3,350	Dextran 10	25	7.3	16.0
2			30	8.2	17.0
3	PEG 10,000		25	5.6	15.0
4			30	6.8	15.9
5	PEG 3,350	Dextran 100	15	3.5	8.2
6			20	4.0	11.1
7			25	4.9	13.5
8			30	6.8	15.0
9	PEG 10,000		15	5.4	10.0
10			20	5.4	12.1
11			25	6.1	14.1
12			30	7.0	15.5
13	Dextran 70	Ficoll 400	15	12.8	11.0
14			20	13.5	11.6
15			25	15.2	13.1
16			30	16.3	14.0
17	PEG 10,000		15	5.5	12.7
18			20	6.0	15.0
19			25	7.0	16.0
20			30	7.5	18.0

Systems were designed as described in materials and methods section. For all systems, V_R_ and pH was kept constant at 1.0 and 7.0, respectively. TLL, V_R_ and the composition of each system were estimated using the binodal curves presented by Zaslavsky and Croll *et al*. Systems highlighted in gray (i.e., 1, 3, 7 and 14) were selected for parameter optimization with the addition of NaCl.

**Fig 1:**
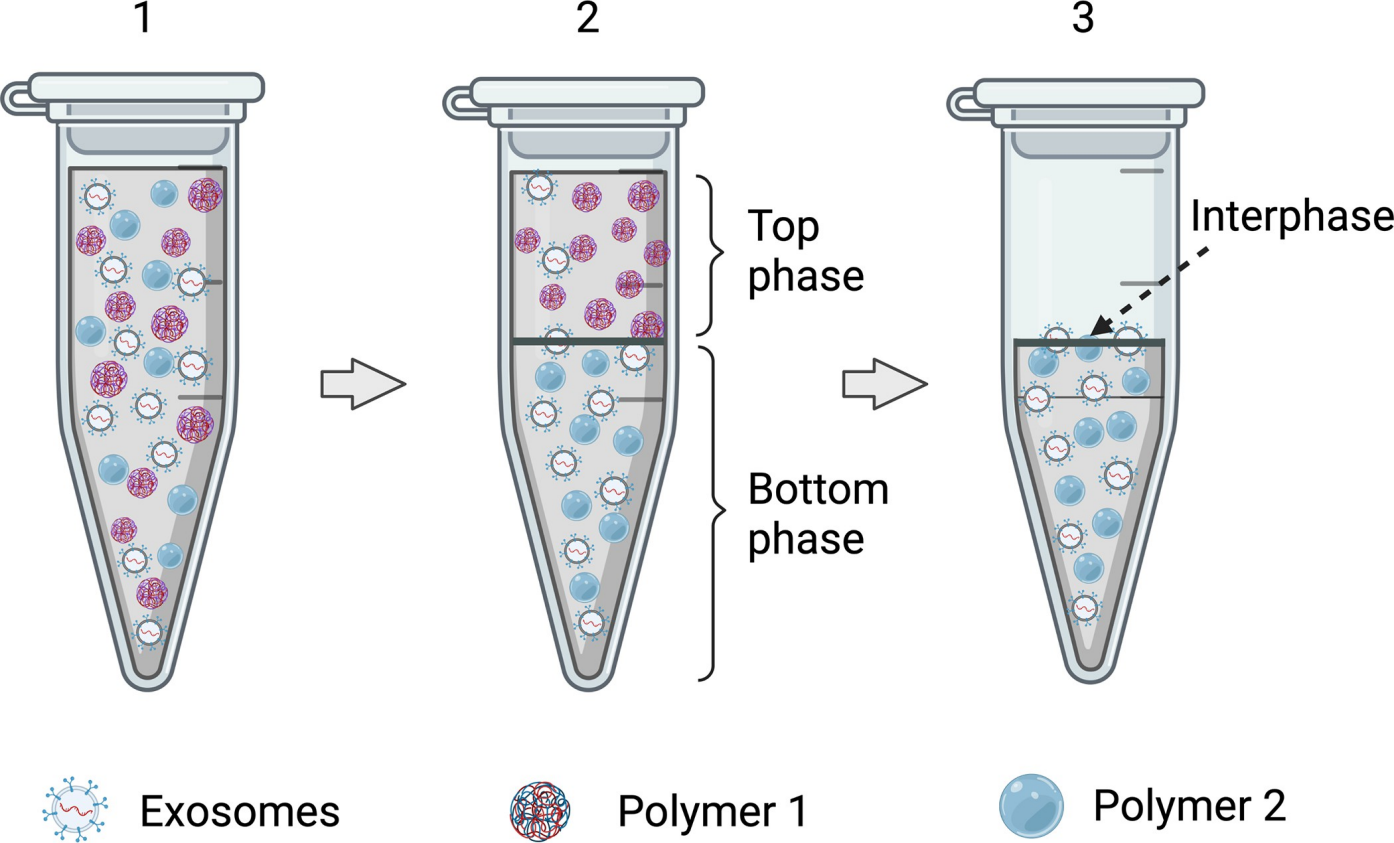
Key steps in the ATPS exosome purification process. 1) Mixture of polymer solutions and samples (i.e., exosomes or contaminants separately); 2) phases formation and exosome separation after vortex and centrifugation steps; 3) recovery of top phase composed mainly of polymer 1 for further quantification of isolated samples. The protein recovery yield for the exosomes and contaminant samples was estimated for both phases, considering the interphase as part of the bottom phase composed of mainly of polymer 2. Figure created using Biorender.com.

The key steps in the ATPS exosome purification process are represented in Fig 1. The systems that presented a better performance in the separation of exosomes from the contaminants were selected for further optimization. These systems were supplemented with neutral salt (NaCl) to reach a total final concentration of 25 mM and then analyzed under the same procedure as described in the next section.

### Protein quantification of isolated samples

After stabilization, 50 μL were carefully taken from the top and bottom phases with a micropipette in each system for total protein quantification. Protein content in each phase was determined by measuring absorbance at 280 nm in a NanoDrop® 1000 Spectrophotometer (Thermo Scientific, USA), using a previously prepared calibration curve (ε = 0.0005, R^2^ = 0.9983) with Bovine Serum Albumin as standards at concentrations of 25, 50, 100, 250, 500, 750, 1000 and 1500 μg/mL. It is important to remember that because of their characteristics, exosomes present embedded proteins in their surface. After verification of exosome isolation according to the procedures previously presented and according to the exoELISA kit, 2 mg/mL of protein concentration are equivalent to 1x10^10^ particles/mL allowing the calculation of the number of exosomes isolated from the CaCo2 cell culture [8, 33]. Protein concentration was used to analyze results reported in this work. The volumes of the top and bottom phases in each replicate were estimated visually using graduated tubes to verify the V_R_ in each system. The protein recovery yield for the exosome and contaminant samples was estimated for both phases, considering the interphase as part of the bottom phase. Partition coefficient (K_P_) was calculated as the protein concentration ratio between the phases [9, 23]. All measurements were performed at least three times.

### Analytical and descriptive procedures

At least three replicates for each system were made for all experiments and measurements. Data reported in this work is expressed as the average of the results with its corresponding standard error, which was calculated as the standard deviation divided by the square root of the number of replicates.

The first part of the fractionation analysis was done separately for exosome and cell-culture media contaminants by comparing the effect of system design parameters (i.e., polymer composition, molecular weight, and TLL) directly on the corresponding recovery yield of each phase. For ATPS optimization, a qualitative comparison was made based on the results of the natural logarithm of the partition coefficients of both sample types: exosomes, and contaminants.

### Economic evaluation

The economic analysis performed here is based on the use of materials and consumables in a laboratory scale (from 1 mL of cell culture volume to 10 L). All materials and consumables required for cell culturing (e.g., serological pipettes, plates, media, etc.) and for exosome recovery using ATPS (i.e., phase forming chemicals). Using supplier’s information on package sizes and prices, along with minimal material usage in the laboratory, it was possible to calculate the exact production cost per mg of exosomal protein.

To enhance economical calculations, this work analyzed the impact of varying the recovery yield of the ATPS (as all experimental results show variability), the effect of having different materials costs (denoted as a percentage of the current 100% used for the construction of the model ranging from 100% of the total cost to 20%) and the impact of producing at different scales (from 1 mL of cell culture to 10 L). A collection of the materials needed, including their prices, and an example for a 100 mL can be found in S1 Table.

## Results and discussion

### Analysis of exosome fractionation from cell-culture media contaminants

To determine the optimal design ATPS parameters for exosome isolation, twenty different polymer-polymer compositions along with at least two different TLL were studied as depicted in Table 1. As mentioned, the influence of system design parameters upon the partition behavior of exosomes and contaminants was evaluated in a discrete way in which exosomes and contaminant partition was evaluated separately.

### Exosome characterization and protein quantification

Exosome size ranged between 30 and 150 nm according to the methodology used for isolation and analysis. In this work, exosomes were isolated from exosome-enriched culture media using conventional ultracentrifugation preparation method. From the characterization experiments, particle size distribution and zeta potential results were consistent with exosome characteristics in previous published works [8, 29, 33, 34]. The diameter size of the obtained exosomes ranged from 40 nm to 235 nm with an average size of 152.00 ± 55.13 nm, well within the reported values [8, 29]. The average zeta potential of the particles was -29.00 ± 4.10 mV, which is expected because of the negatively charged phospholipid membrane of the particles [30].

Most importantly, this obtained zeta potential value acts as an indicator of particles stability in colloidal dispersions [35], which is influenced by the net surface charge. In this sense higher negative Z-potential values indicate a larger stability for hydrophobic particles [36].

An absorption-based quantitation method was chosen in this study to track the partitioning preference of the exosomes by calculating embedded protein concentration in the nanoparticle surface and, on the other hand, the contaminant proteins from the culture media. Since proteins are among the most abundant exosome components, it has been reported that embedded protein concentration in exosomes is directly related to the number of vesicles in the sample as demonstrated by simultaneous exosome quantification by dynamic light scattering (DLS) and protein concentration using the Bradford method [37]. The partition behavior of exosomes and contaminants on each ATPS is shown in Table 2.

**Table 2:** Effect of polymer composition, molecular weight (MW) and tie-line length (TLL) upon the partition coefficient, recovery yield and selectivity of exosomes and contaminants in each aqueous two-phase system.

				Exosomes			Contaminants		
				Recovery Yield (% w/w)			Recovery Yield (% w/w)		
System	Polymer 1	Polymer 2	TLL	Top Phase	Bottom Phase	ln (K_P_)	Top Phase	Bottom Phase	ln (K_P_)
**1**	PEG 3,350	Dextran 10	25	39.35 ± 09.62	60.65 ± 00.81	-0.43 ± 0.02	53.28 ± 07.56	46.72 ± 06.83	0.13 ± 0.00
**2**			30	44.97 ± 00.28	55.03 ± 04.64	-0.20 ± 0.03	44.37 ± 04.96	55.63 ± 13.84	-0.23 ± 0.01
**3**	PEG 10,000		25	42.68 ± 01.58	57.32 ± 03.41	-0.29 ± 0.01	15.65 ± 11.76	84.35 ± 04.05	-1.68 ± 0.01
**4**			30	03.50 ± 00.00	96.50 ± 29.55	-3.32 ± 0.00	20.06 ± 05.13	79.94 ± 02.58	-1.38 ± 0.01
**5**	PEG 3,350	Dextran 100	15	03.50 ± 16.13	96.50 ± 08.83	-3.32 ± 0.01	18.08 ± 01.10	81.92 ± 00.00	-1.51 ± 0.00
**6**			20	34.94 ± 00.00	65.06 ± 02.29	-0.62 ± 0.00	20.46 ± 00.06	79.54 ± 00.59	-1.36 ± 0.02
**7**			25	02.87 ± 00.00	97.13 ± 16.46	-3.52 ± 0.00	22.92 ± 00.54	77.08 ± 07.36	-1.21 ± 0.03
**8**			30	08.22 ± 00.00	91.78 ± 09.36	-2.41 ± 0.00	11.36 ± 00.88	88.64 ± 00.57	-2.05 ± 0.00
**9**	PEG 10,000		15	16.13 ± 00.00	83.87 ± 00.00	-1.24 ± 0.00	15.87 ± 00.60	84.13 ± 01.18	-1.67 ± 0.01
**10**			20	01.56 ± 01.30	98.44 ± 07.00	-3.74 ± 0.02	08.55 ± 01.07	91.45 ± 02.26	-2.37 ± 0.01
**11**			25	01.42 ± 00.00	98.58 ± 24.14	-4.05 ± 0.00	01.17 ± 01.17	98.83 ± 05.26	-4.43 ± 0.01
**12**			30	01.34 ± 04.55	98.66 ± 02.53	-4.30 ± 0.01	00.76 ± 00.76	99.24 ± 08.67	-4.87 ± 0.02
**13**	Dextran 70	Ficoll 400	15	01.76 ± 00.00	98.24 ± 28.21	-4.43 ± 0.00	38.28 ± 00.85	61.72 ± 02.67	-0.48 ± 0.01
**14**			20	15.60 ± 03.90	84.40 ± 00.00	-1.83 ± 0.00	62.73 ± 02.36	37.27 ± 03.42	0.52 ± 0.00
**15**			25	62.49 ± 04.19	37.51 ± 05.59	0.51 ± 0.00	73.29 ± 01.58	26.60 ± 01.27	1.01 ± 0.00
**16**			30	65.35 ± 00.00	34.65 ± 03.69	0.63 ± 0.00	65.76± 00.09	34.24 ± 00.19	0.65 ± 0.01
**17**	PEG 10,000		15	00.79 ± 00.00	99.21 ± 00.58	-5.10 ± 0.00	01.87 ± 01.87	98.13 ± 29.89	-3.96 ± 0.03
**18**			20	03.50 ± 00.00	96.50 ±13.93	-3.32 ± 0.00	01.82 ± 01.82	98.18 ± 00.00	-3.99 ± 0.00
**19**			25	02.69 ± 00.00	97.31 ± 09.48	-3.59 ± 0.00	02.79 ± 01.94	97.21 ± 00.00	-3.55 ± 0.00
**20**			30	06.92 ± 06.92	93.08 ± 06.16	-2.60 ± 0.00	08.38 ± 00.00	91.62 ± 01.94	-2.39 ± 0.00

Selected systems for further optimization are highlighted in gray. For all systems, VR and pH were kept constant at 1.0 and 7.0, respectively.

### Partition behavior of exosomes

In general, this research shows that exosomes have a significantly higher preference to the bottom phase than to the top phase since this was observed in 18 out of the 20 systems under study, while recovery yields ranged from 55 ± 4.64% to 99 ± 0.58% in this same phase. However, changes in system design parameters must be studied to find alternatives to manage exosome partition behavior and find the best conditions for its separation from culture-medium contaminants.

The first 4 systems (see Table 2) were evaluated at TLL values of 25% and 30%. It is important to mention that for these polymer combinations, lower TTL values, as presented for the rest of the systems, cannot be generated because of the high polymer concentrations needed to obtain the biphasic system. Nonetheless, in the PEG 3,350—Dextran 10 systems, the increase of TLL by 5%, represented a decrease in the bottom Dextran-rich phase partition of exosomes from 60.65 ± 0.81% to 55.03 ± 4.64%; whereas in the PEG 10,000—Dextran 10 systems this same TLL increment enriched exosomes in the bottom Dextran-rich phase and increase recovery yield from 57.32 ± 3.41% to 96.5 ± 29.55%. Considering that the main difference between these two sets of ATPS (i.e., systems 1–2 and 3–4) is the increment in polymer molecular weight (MW), this parameter has a particular effect in this behavior since the higher polymer concentrations at the PEG-enriched phase decrease the available free volume between polymer molecules caused by the increment of PEG chain lengths rendering less volume for exosomes to partition towards this phase [18, 38]. Other works where similar polymers have been used, suggest that exosomes present a higher preference towards the Dextran-rich phase [9, 26, 28] which according to these results is true for systems where large PEG MW are used and free volume becomes a factor affecting partition. The rest of the systems (5 to 20; see Table 2) were evaluated at 4 different TLL values; 15, 20, 25 and 30% w/w. PEG 3,350 –Dextran 100 and PEG 10,000 –Dextran 100 systems presented higher exosome partition towards the bottom Dextran-rich phase. In the case of the PEG 3,350 –Dextran 100 systems, the highest protein recovery yields (~97%) were obtained at TLL 15% and 25% with a negligible difference between them (0.63%; see systems 5 and 7 in Table 2). For the PEG 10,000 –Dextran 100 systems, no significant difference between the highest recovery yields (<98%) was observed between the TLL 20, 25 and 30% w/w systems. Considering these ATPS sets (i.e., systems 5–8 and 9–12), the best exosome recovery yield increased only in around 1% when PEG MW increased. Therefore, results show that the partition of exosomes in these sets is mostly independent from TLL and PEG MW and is rather affected by polymer hydrophobicity. In this sense, PEG polymers present a relatively high hydrophilicity due to the availability of polar -OH moieties in its chemical structure but at the same time are less hydrophilic than Dextran. Furthermore, the hydrophobic character of PEG and the hydrophilic character of Dextran increase in proportion to their MWs. Therefore, in these systems, exosomes present a higher partition preference towards the Dextran-rich phase because of the high hydrophilicity this polymer presents, and the increments of hydrophobicity observed in PEG as the selected MWs increase rather than by increments in their relative concentrations. Besides, this separation behavior is also expected because exosomes present extremely hydrophilic surfaces [19, 36]. It is important to mention that from this set of systems, the one evaluated at TLL 20% w/w (i.e., system 6) presented a different behavior with a recovery yield of 65.06 ± 2.29% in the bottom phase, which is around 29% lower than the rest (< 91%). This particular polymer composition showed that the separation could be affected by other variables in the process such as temperature or separation time that might have an effect in the load capacity of Dextran-rich phase. Nonetheless, this system was considered as an outlier which did not behave under the same tendencies as the rest of the PEG 3,350 –Dextran 110 systems.

Further on, it is evident that in the Dextran 70 –Ficoll 400 system, exosome partition behavior is dependent on TLL. As TLL increases, the partition behavior shifts towards the top Ficoll-rich phase. At TLL 15 and 20% w/w exosome recovery yields are higher at the bottom Dextran-rich phase, 98.24 ± 28.21% and 84.40 ± 0.00%, respectively, while at 25% and 30% w/w the molecule has a higher preference to the top Ficoll-rich phase with recovery yields of 62.49 ± 4.19% and 65.35 ± 0.00% respectively (see systems 13 to 16 in Table 2). This behavior has been previously explained by Nisslein *et al*. (2020) as an effect originated from the chemical similarity between polymers. Both Dextran and Ficoll are sugar-based polymers with a similar distribution of polar and non-polar groups. Moreover, since an increment in TLL causes an increase in polymer concentration in each phase (see Table 1), which in turn, decreases the free volume available for protein partition and modifies the density of both phases and surface tension at the interface [23], changes in the partition behavior of nanoparticles are expected. As a result, at lower polymer concentrations, the systems present lower surface tension allowing particles to move through the bottom Dextran-rich phase or to be trapped at the interphase [9]. Also, since the amount of Dextran 70 is higher than Ficoll 400 in these system sets (i.e., 13 to 16), when the weight composition of polymers increases, the available free volume in the bottom phase is not enough to accommodate the exosomes [23].

The PEG 10,000—Ficoll 400 systems presented a decrease in exosome partition towards the bottom phase while TLL values increased (see systems 17 to 20 in Table 2). Their recovery yield decreased from 99.21 ± 0.58% to 93.08 ± 6.16%, and the highest yield was obtained at the lowest TLL value. This polymer-polymer ATPS present a high-density environment, in which PEG is predominantly found in the top PEG-rich phase with a larger hydrophobic character than Ficoll [18]. This behavior is consistent with other studies, in which hydrophilic proteins and particles have been found to prefer the bottom Ficoll-rich phase in PEG-Ficoll systems [24, 39]. As expected, the exosomes tended to move towards the less hydrophobic phase, while increments in TLL affected free volume in a similar manner as in the previously described cases.

It is relevant to state that all systems achieved a higher isolation yield in the bottom phase than the estimated yield obtained with UC from culture media, which is reported to be between 5% and 25% [9]. In this regard, other studies have compared their ATPS yields with ultracentrifugation results. For example, Shin (2015) *et al*., have reported exosome recovery efficiencies in DEX-rich phases to be 4 times higher than that achieved using UC. The same research group of Shin (2018) *et al*., has also reported a recovery efficiency approximately 14 times higher than what was achieved by two UC steps.

### Partition behavior of cell-culture media contaminants

The purpose of this work is to propose an ATPS-based strategy for the recovery of exosomes from the contaminants found in cell-culture media, thus the study of the partition behavior of said contaminants should also be performed. On its part, protein contaminants in the culture media from which exosomes are isolated were also evaluated in the same ATPS to determine their partition behavior.

Contaminants exhibited a similar partition behavior to exosomes with a marked preference for the bottom phase in 15 out of the 20 systems studied. In the case of PEG 3,350—Dextran 10 systems, the increase of TLL by 5% w/w, increased the bottom phase preference of the contaminants from 46.72 ± 6.83% to 55.63 ± 13.84%. These results suggest that their separation is dependent from TLL. However, protein contaminants are much smaller than exosomes, so they occupy lower volumes in the system making them able to remain at the top PEG-rich phase with similar recovery efficiency while increasing polymer concentration. PEG 10,000—Dextran 10 systems also enriched contaminants in the bottom Dextran-rich phase with a higher recovery yield at the lowest TLL. At a lower polymer concentration, the interfacial tension is also lower, which allows the migration of biomolecules through the interphase easily [9]. From these systems, it could be assumed that the recovery yield is affected by TLL and polymer MW.

On their part, PEG 3,350 –Dextran 100 and PEG 10,000 –Dextran 100 systems (see systems 5–8 and 9–12 in Table 2) showed an increase in the recovery yield from 81.92 ± 0.00% to 88.64 ± 0.57% and 84.13 ± 1.18% to 99.24 ± 8.67%, respectively, while TLL increased. Protein contaminants are hydrophilic and are expected to present a higher preference towards the most hydrophilic phase, that in this case is the Dextran-rich phase [18]. Increases in TLL which, as it has been said, represent increments in the polymer concentration at any given phase increase as well as the hydrophilic or hydrophobic character of the phases promoting these types of interactions between the phase-forming chemicals and the solutes.

Contaminants tested in the PEG 10,000—Ficoll 400 systems showed a significant high preference towards the bottom Ficoll-rich phase (see systems 17–20 in Table 2). The increment in TLL from 15% to 30% w/w caused a decrease in the recovery yield from 98.13 ± 29.89% to 91.62 ± 1.94%. As known, Ficoll is a high MW polymer and an increase in TLL might cause possible saturation of the Ficoll-rich phase, while it also affects density and surface tension at the interphase which probably causes entrapment of molecules in the interphase [24].

### Optimization of ATPS systems via salt addition

From the previous analysis, it is evident that the highest exosome recovery yields were achieved at the bottom phase in all systems, but this was also true for contaminants. Considering the need to find a system in which both exosomes and contaminants were partitioned mainly towards different phases, further analysis were performed to find and later optimize those alternatives.

### Coefficient partition analysis and system selection for optimization

**Fig 2:**
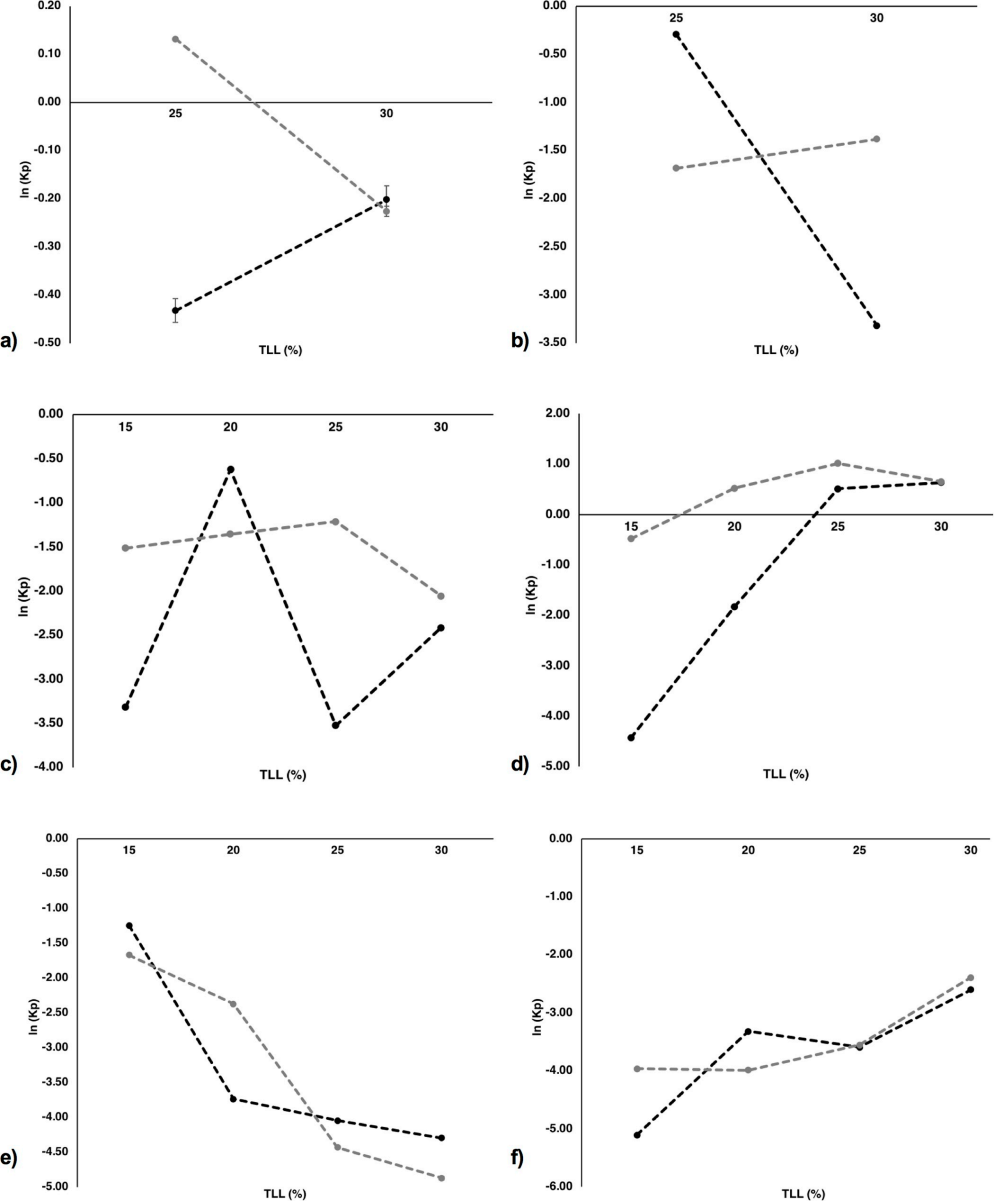
Effect of TLL (%) w/w on ln K_P_ of exosomes and contaminants fractionation in aqueous two-phase systems. Ln K_P_ values of exosomes () and contaminants () are shown for each system: a) PEG 3400 g mol -1—Dextran 10 g mol ^-1^, b) PEG 10000 g mol ^-1^—Dextran 10 g mol^-1^, c) PEG 3400 g mol -1—Dextran 100 g mol ^-1^, d) Dextran 70 g mol-1—Ficoll 400 g mol ^-1^, e) PEG 10000 g mol -1—Dextran 100 g mol ^-1^ and f) PEG 10000 g mol -1—Ficoll 400 g mol ^-1^ ATPS; a) and b) were tested at TLL 25% and 30% w/w, and c-f) were tested at TLL 15%, 20%, 25% and 30% w/w. VR and pH were kept at 1 and 7, respectively.

In doing so, the natural logarithm of the partition coefficients (ln(K_P_)) of both exosome and contaminants were analyzed with respect to TLL, because even when all systems are different in polymer composition they were evaluated considering the same TLL values. The effect of TLL on ln(K_P_) of exosomes and contaminants in each ATPS is depicted in Fig 2. This arrangement allows us to identify the optimal parameter selection for those systems which present a potential exosome isolation from their contaminants. A more negative value for ln(K_P_) indicates that the studied particle prefers the bottom phase, while a more positive value portrays a preference towards the top phase. As it is seen in Fig 2A, for the PEG 3,350—Dextran 10 systems the largest negative ln(K_P_) value (-0.43 ± 0.02) for exosomes was obtained at TLL 25% w/w while this system also presented the highest positive ln(K_P_)value for contaminants (0.13 ± 0.00) making it a good candidate for further optimization. In the case of the PEG 10,000 –Dextran 10 systems, illustrated in Fig 2B, all ln(K_P_) values were negative. However, at TLL 25% w/w the smallest negative ln(K_P_) value was of -0.29 ± 0.01 for exosomes, which was almost 6 times less negative than the ln(K_P_) value obtained for the contaminants (-1.68 ± 0.01) at the same TLL. A similar behavior was seen for the PEG 3,350 –Dextran 100 TLL 25% system found in Fig 2C. In this case, the smallest negative ln(K_P_) value for the contaminants was of only -1.21 ± 0.03 but it was almost 3 times less negative than the ln(K_P_) value for the exosomes (-3.52 ± 0.00). On its part, the Dextran 70—Ficoll 400 system at TLL 20% w/w (Fig 2D) showed a positive ln(K_P_) value (0.52± 0.00) for the contaminants and a negative value (-1.83 ± 0.00) for exosomes, presenting a good opportunity to try to enhance this difference. All these last systems (i.e., systems 1, 3, 7 and 14; highlighted in Table 2) are therefore considered potential candidates for further optimization. In this sense, even when some of the ln(K_P_) values of the selected systems are negative for both exosomes and contaminants, an improvement in the partition preference of the different species by the addition of a neutral salt like NaCl can be expected. This because both Na^+^ and Cl^-^ ions exert an effect in polymer hydrophobicity and could promote electrostatic interactions that can alter partition effects [36]. In this regard, for example, Hernandez-Vargas (2019) *et al*., optimized their ATPS by adding NaCl and reported that the salt addition proved to be a major provider of favorable conditions for the separation of their studied molecules in the systems [40].

It is important to mention that the partition behavior observed in the PEG 10,000—Dextran 10 and PEG 10,000—Ficoll 400 systems can be found in Fig 2E and 2F, respectively. However, in both sets of systems, exosomes and contaminants present a very similar partition behavior towards the bottom phase based on the ln(K_P_) analysis. It is also clear that as TLL increases in the PEG 10,000 –Dextran 10 systems (Fig 2E) partition of both exosomes and contaminants towards the bottom Dextran-rich phase increases. On the other hand, in the PEG 10,000 –Ficoll 400 systems (Fig 2F) the partition of both species towards the bottom Ficoll-rich phase proportionally decreases. Nonetheless, none of these systems will be considered for further optimization since partition behaviors of both exosomes and contaminants are always of the same magnitude regardless of TLL changes and remain mostly in the bottom phase.

In summary, from the ln(K_P_) analyses for both exosomes and contaminants the 4 systems that presented the best exosome isolation parameters under initial conditions were selected for further partition optimization with the addition NaCl to reach a total final concentration of 25 mmol L^-1^. It is well known that salt addition is a reliable way to manipulate partition behavior when using ATPS as primary recovery stage and that this effect can be observed even at low salt concentrations [40].

### Optimized ATPS analysis

The effect of NaCl on ln(K_P_), recovery yield and selectivity for exosomes and contaminants in the selected ATPS are reported in Table 3. From the selected candidates, only two NaCl-enriched systems improved the separation behavior of the solutes, these systems were PEG 3,350—Dextran 10 and PEG 10,000—Dextran 10 systems both at TLL 25%, (i.e., 1 and 3), improved the separation behavior of the solutes. The addition of salt in these ATPS modified the molecules preference shifting exosome partition towards the top phase. The addition of NaCl to the other systems caused an increased bottom phase preference for both exosomes and contaminants in all selected systems.

**Table 3:** Effect of polymer composition, molecular weight (MW) and tie-line length (TLL) upon the partition coefficient, recovery yield and selectivity of exosomes and contaminants in each aqueous two-phase system to reach a final concentration of 25 mM NaCl.

				Exosomes				Contaminants		
				Recovery yield (% w/w)				Recovery yield (% w/w)		
System	Polymer 1	Polymer 2	TLL	Top Phase	Bottom Phase	ln (K_P_)	Top Phase	Bottom Phase	ln (Kp)	Selectivity
1	PEG 3,350	DEX 10	25	63.29 ± 9.17	36.71 ± 5.65	0.54 ± 0.00	40.70 ± 2.16	59.30 ± 10.15	-0.38 ± 0.02	2.51 ± 0.02
3	PEG 10,000		25	85.16 ± 4.35	14.84 ± 2.42	1.75 ± 0.01	31.79 ± 0.58	68.21 ± 1.87	-0.76 ± 0.01	12.31 ± 0.01
7	PEG 3,350	DEX 100	25	3.20 ± 0.00	96.80 ± 27.60	-3.24 ± 0.00	4.81 ± 0.92	95.19 ± 5.36	-2.91 ± 0.02	0.72 ± 0.02
14	DEX 70	FICOLL 400	20	13.61 ± 4.50	86.39 ± 9.30	-1.99 ± 0.01	25.18 ± 1.26	74.82 ± 5.18	-1.09 ± 0.01	0.41 ± 0.01

For all systems, VR and pH were kept constant at 1.0 and 7.0, respectively.

**Fig 3:**
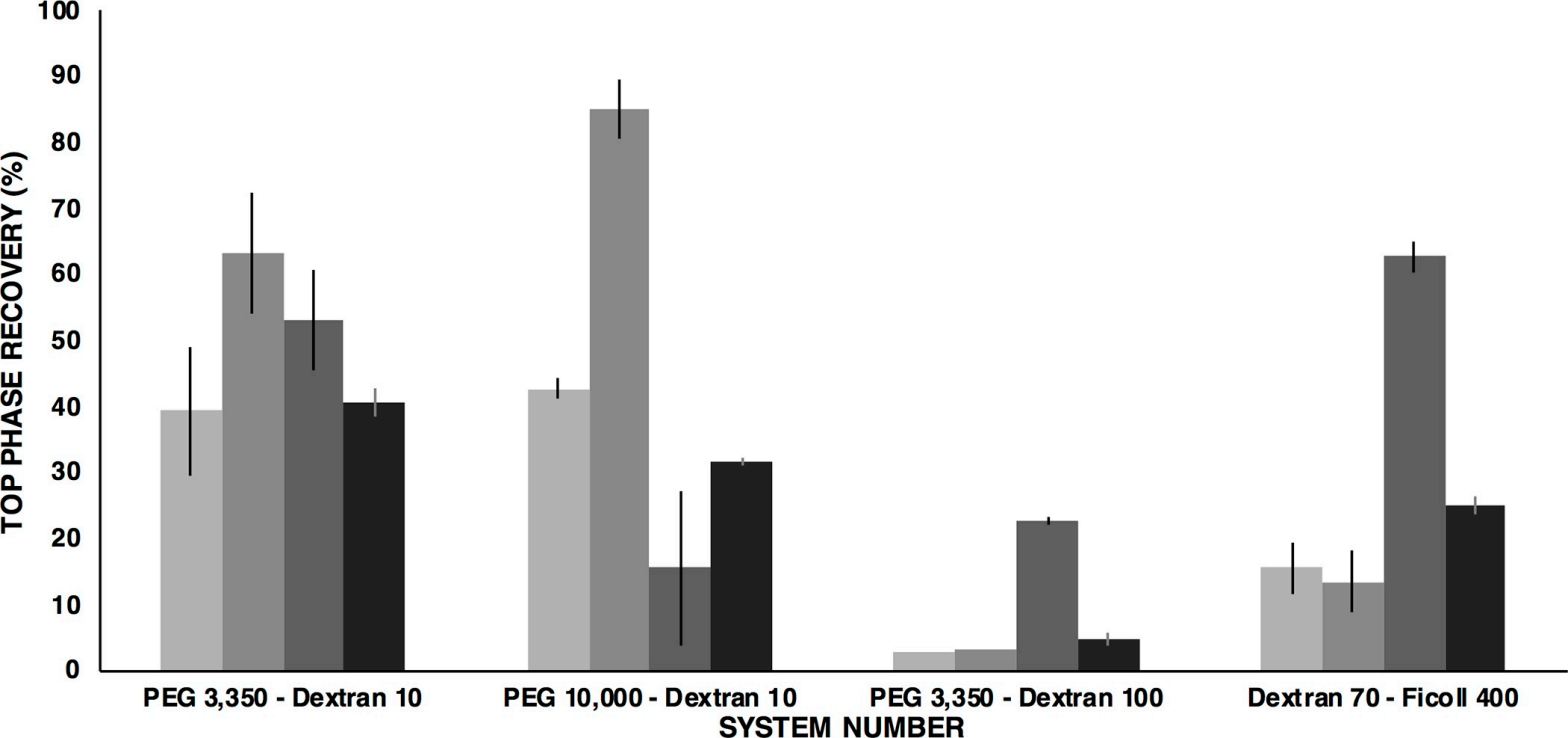
Effect of NaCl upon top phase recovery yield of exosomes and contaminants in the selected systems. The height of each bar represents the top phase recovery percentages for exosomes (∎) and contaminants (∎) under initial conditions, and after 25 mmol L^-1^ NaCl addition (∎) and (∎), respectively.

Fig 3 compares the top phase recovery yields of exosomes and contaminants of previous results reported in Table 2 with the ones after NaCl addition found in Table 3.

**Fig 4:**
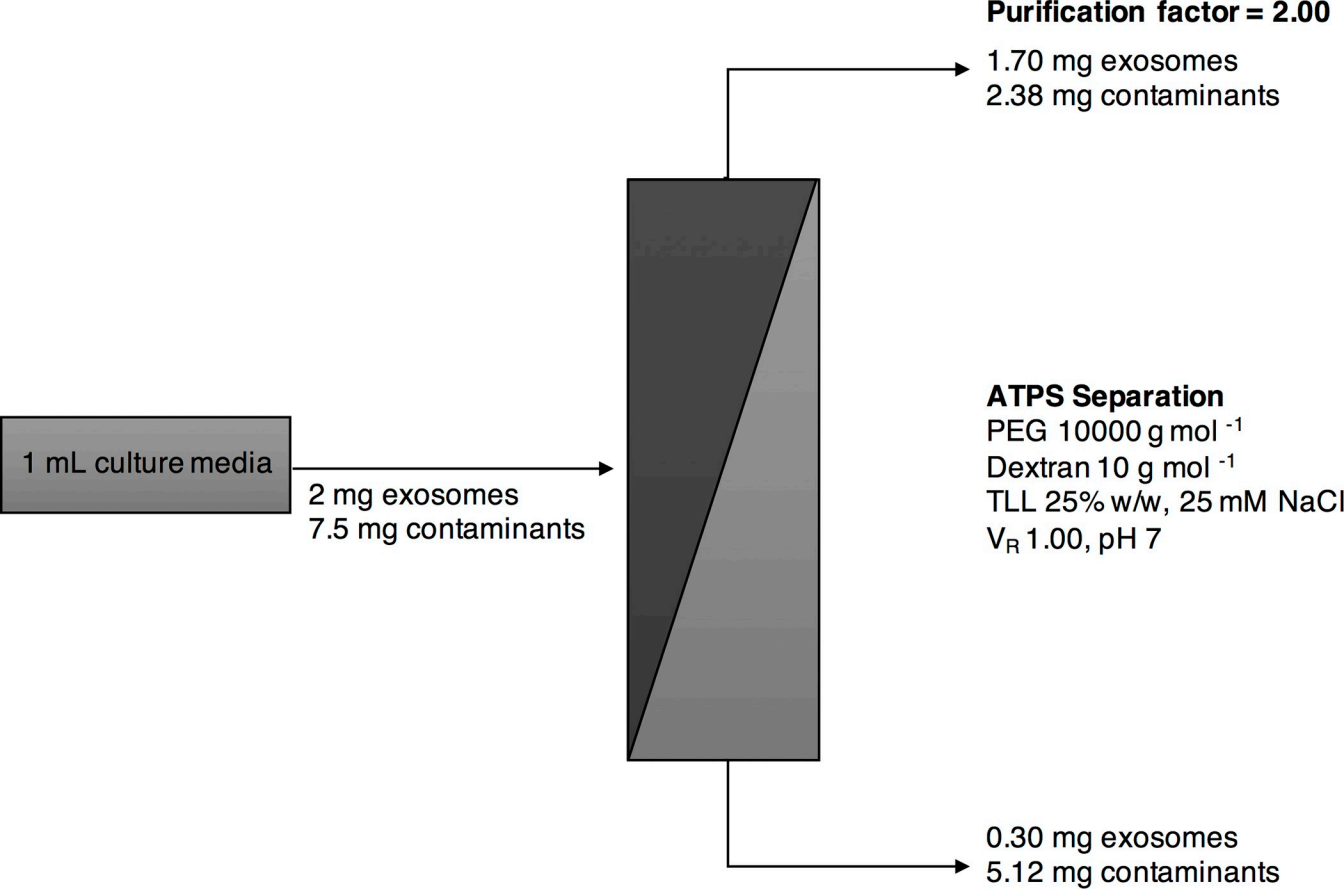
Simplified scheme for the optimized ATPS strategy to selectively fractionate a complex sample. The proposed strategy suggests that after the optimization of the system PEG MW 10000 g/mol—DEXTRAN MW 10000 g/mol, TLL 25% w/w, VR 1.00, pH 7, this ATPS could separate the culture media containing exosomes and contaminants at opposite phases. The recovery yields shown are theoretical according to the previous reported results in this work.

As noted, NaCl has a significant impact upon the intrinsic ionic strength of each system because of the increment in ion concentration. The presence of ions, which are also partitioned, allows ionic interactions with the molecules in the system [40]. Exosomes prefer the most hydrophilic phase (as noted in systems 1 and 3) after NaCl addition. These systems showed better isolation efficiency of exosomes in the top phase from the contaminants that remained in the bottom phase resulting in higher recovery yields. However, it is believed that the PEG 10,000 –Dextran 10 at TLL 25% w/w ATPS represents the best option for this task since it shows the best isolation yield and higher selectivity. Therefore, this last system is proposed for further analyses with complex samples such as culture media from the CaCo2 cell line containing exosomes and proteins. In this line, Fig 4 presents the expected mass balance in the ATPS for the optimized system, which was calculated assuming that the partition behavior of a complex exosome–contaminant mixture will perform similarly as the obtained discrete results reported above in this work. As a matter of fact, it has been found that after discrete analysis, sample mixtures tend to behave in a similar manner when using the same ATPS conditions [28, 39, 40]. Moreover, to enhance purification of exosomes from host cell proteins an additional stage of ATPS can be implemented as previously reported for complex samples [9, 25, 26].

Following this strategy, a purification factor of 2 in the top PEG-rich phase can be achievable in the top PEG-rich phase. This purification factor might be considered low for typical ATPS results, but it directly depends on product type and sample complexity. Since exosomes can be regarded as very high added-value product, this result might be still considered as an attractive scenario. Furthermore, if the system is performed at least twice by the addition of fresh Dextran-rich bottom phase, the amount of protein contaminants would be significantly reduced in the top phase. This additional step could minimize protein impurity with no significant change in exosome concentration. This has also been previously performed since, for example, Shin, *et al*. (2018), improved the recovery efficiency of extracellular vehicles (EVs) in the bottom Dextran phase after replacing three times the PEG phase, reducing the number of proteins contaminants, and recovering approximately 100% of EVs [26].

By the natural composition of polymers, it is expected that the integrity of the exosome and their physicochemical properties would remain intact after this primary recovery stage. In addition to the fact that these nanoparticles partition better in these systems, preliminary results suggest an effect on the size fractionation of exosomes (data not shown). In general, these results confirmed that exosome fractionation is possible by using aqueous two-phase systems with high particles recovery and that the addition of NaCl provided favorable conditions for the separation.

### Economic evaluation

After the design of the process presented in Fig 4, an economic evaluation was performed to determine the production cost using the proposed process (and the selected ATPS). One of the main considerations in this evaluation was to only include costs associated with materials, as exosome production is performed at laboratory scales. Using the data in S1 Table, a model was created that had as variable the production scale, for which different scenarios were tested including 1 mL, 10 mL, 100 mL, 1 L and 10 L. All calculations were performed based on the following Eq (1) [41], including the impact of different variables.


$$\frac{Production\;Cost}{mg\;of\;Exosome\;protein}=\frac{\sum_{i=1}^{n}\left(\frac{Use\;of\;{Material}_{\;i}}{Batch}*\frac{Price\;of\;{Material}_{\;i}}{Unit\;of\;{Material}_{\;i}}*Materials\;Multiplier\right)}{\frac{Mass\;of\;Exosome\;Protein}{Volume\;of\;Cell\;Culture}*ATPS\;Recovery\;Yield*Production\;Scale}$$ (1)

**Fig 5:**
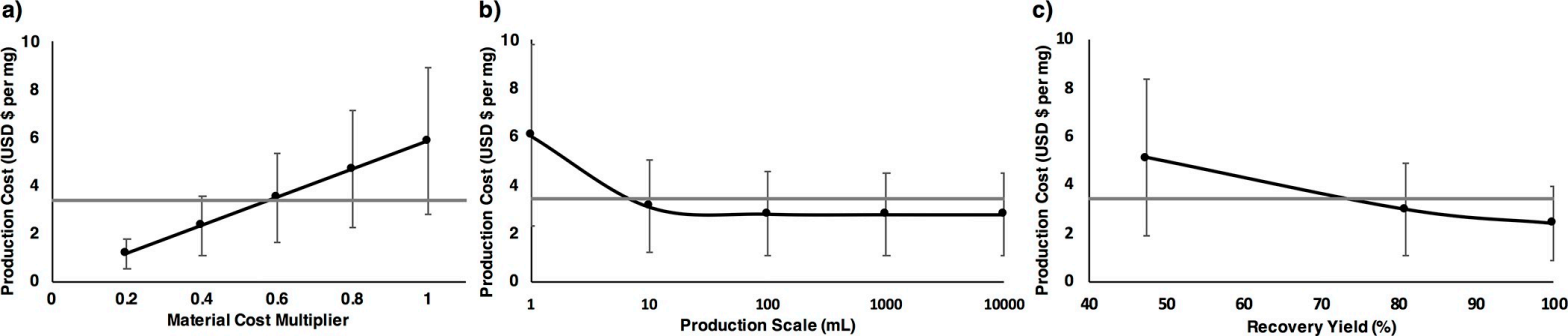
Results for the economic evaluation. a) shows the impact of decreasing the prices of materials from the base value (multiplier = 1) up to a fifth (multiplier = 0.2), b) shows the impact of using different production scales, and c) the impact of varying the recovery yield. Grey line depicts the benchmark value of using a commercial method for exosome purification (USD $3.42 per mg of exosome protein).

Using the production titer from Fig 4 (i.e., 2 mg of exosomes per mL of cell culture), it is possible to integrate recovery yields (80.85% ± 33.48% for system 3) from the ATPS to determine the impact that variation found in previous sections has on the production cost. For the analysis of the recovery yield, a base scenario was constructed using the average of the yield (80.85% as seen in Fig 3) while a worst scenario was considered using the average minus one standard deviation. For the best scenario, the average plus standard deviation will be higher than 100% recovery, so this value was selected as the limit and used. Additionally, the current materials/consumable prices were obtained from suppliers directly, but it is possible to acquire bulk quantities that can qualify for a potential discount. To capture this, a multiplier for materials was included to understand how a decrease in prices from materials will affect the results. The multipliers included 1 (for a 100% of the current price), 0.8, 0.6, 0.4, and 0.2 (representing a 20% of the current price or an 80% discount). Production costs were calculated at all possible combinations for the different recovery yields, material multipliers, and production scales (a total of 75 combinations). Results for each variable are presented in Fig 5. With the current developments and the analysis performed here, production costs calculated have an average of USD $3.50 per mg of exosome proteins (median of USD $2.85 per mg) and can range from a minimum of USD $0.64 to a maximum of USD $14.7 per mg.

To determine the relevance of each parameter, a multiple linear regression was constructed [37] and results are presented in Table 4. Considering the p-values obtained for the three variables, the most significant variable is the materials multiplier. This makes the prices of the materials highly critical for the process. This is to be expected as the model is based mainly on materials, but its relevance is reflected furthermore in a subsequent contrast. Inversely, the production scale is a variable that is barely significant (depending on the significance level decided). This behavior is a consequence of doing a scale-out model instead of a scale-up. Research published in scale-up makes the production scale highly significant for the behavior of production costs, as the scale increases, the amount of product per batch can increase linearly, but the production cost does not and tend to stabilize, when combining both to generate a production cost per unit mass of product, the production cost decreases when the scale increases [42]. The same general behavior can be seen here (Fig 5b), but less accentuated and, ultimately, barely significant.

**Table 4:** Multiple linear correlation between material cost multiplier, production scale, and recovery yield.

	Coefficients*	P-value
Intercept (βo)	4.226713	7.41E-07
Material Cost Multiplier (β1)	5.849637	2.02E-13
Production Scale (mL) (β2)	-0.00011	0.027914
Recovery Yield (%) (β3)	-5.24878	2.88E-08

*Regression equation has the form

$Production\;Cost\;[USD\;\$\;per\;mg]=\beta_{0}+Material\;Cost\;Multiplier\times \beta_{1}+Production\;Scale\;[mL]\times \beta_{2}+Recovery\;Yield\;[\%]\times \beta_{3}$

Lastly, a commercial product (Thermo Fisher Cat. No. 4478359) was used as a benchmark with a commercial price of approximately USD $380 per 50 mL of reagent (USD $7.60 per mL). Fig 4 includes a horizontal line to depict the price needed per mg of recovered Exosome proteins (USD $3.42 per mg). This last analysis is critical to determine the current state of this ATPS development. For materials costs, when it has a 0.4 multiplier (equivalent to a 60% discount), there are a minor number of scenarios where the production cost using ATPS is above the use of a commercial product. On the other hand, production scale and recovery yield, even at their lowest production cost are still a considerate possibility to obtain a production cost higher than the USD $3.42 benchmark. For this reason, it is critical do work on increasing recovery yield but also in decreasing variability. Moreover, it is important to consider other suppliers for the current production. Lastly, if a commercial scale can be decided, it will be critical to contrast scale-up versus scale-out to determine which option will indeed generate a lower production cost.

## Conclusions

According to the results reported in this study, ATPS design parameters (i.e., polymer composition, MW, and TLL) have a significant effect on the partition behavior of exosomes and contaminants on polymer-polymer ATPS. The analysis of the fractionation of both samples, obtained from CaCo2 culture media, was done in a discrete way proving the efficiency of 4 out of 20 systems to separate our particle of interest to a different phase from the contaminants. In these systems, polymer concentration, hydrophobicity, density, and surface tension between the phases were analyzed as the physicochemical properties that could explain the different effects on the interactions and preference of the studied particles with the polymer phases. It was found in general that exosomes preferred the most hydrophilic phase.

Interestingly, the addition of NaCl provided favorable conditions to 2 out of the 4 selected systems improving the partition behavior of exosomes towards the top phase, while increasing contaminants preference to the bottom phase. However, the PEG 10,000 –Dextran 10 system at TLL 25% w/w, V_R_ 1.0, pH 7.0 and with the addition of NaCl at a final concentration of 25 mM, showed the best isolation yield resulting in an exosome purification factor of 2 in the top phase while most contaminants partitioned to the opposite phase. In this sense, we have confirmed that the ATPS present higher recovery yields than UC (i.e., 5–25% from culture media) with the possibility of eliminating a greater amount of contaminants, which cannot be achieved with a traditional UC method. However, further studies are needed to test different exosome sources using the optimized ATPS reported in this work.

These results are important since the use of polymer-polymer ATPS for primary recovery and purification of exosomes have not been fully explored yet. This isolation technique also represents an attractive and promising method for industrial processes because of its scale-up easiness and potential economic feasibility as it has been shown in the economic analysis performed. Nevertheless, further experiments with exosome-enriched culture media must be conducted in which exosomes and proteins are combined at known concentrations, to rule out other effects in the partition behavior such as protein-protein interactions. The behavior of discrete results reported above are expected to result in similar recovery yields for complex sample analysis. Furthermore, the performance of the selected system could be optimized by studying different NaCl concentrations or replacing the bottom phase with fresh phase, at least twice, to minimize protein impurity in the top phase for efficient downstream analysis.

## Supporting information

S1 Table Data condensate used for model construction. Case scenario included shows the amount and prices needed for the materials to generate 90 mL of cell culturing and its respective recovery using ATPS.
(PDF)
