# Supplementary material for: Characterization and optimization of polymer-polymer aqueous two-phase systems for the isolation and purification of CaCo2 cell-derived exosomes
Source: PLoS One. 2022 Sep 2;17(9):e0273243. doi: 10.1371/journal.pone.0273243 (PMC9439200; doi:10.1371/journal.pone.0273243)
Supplement: S1 Table — Case scenario included shows the amount and prices needed for the materials to generate 90 mL of cell culturing and its respective recovery using ATPS. (PDF) [file pone.0273243.s001.pdf]

# Supporting information

**S1 Table. Data condensate used for model construction.** Case scenario included shows the amount and prices needed for the materials to generate 90 mL of cell culturing and its respective recovery using ATPS.

| Cell Culture | | | |
|---|---|---|---|
| **Item** | **Cost per unit (USD $/g, USD$/mL, or USD $/piece)** | **Amount need for 90 mL of Cell culture (mL, g, or pieces)** | **Cost for 90 mL of Cell Culture (USD $/batch)** |
| Petri Dish | 0.779 | 15 | 11.680 |
| Filtration System (for 500 mL) | 8.198 | 1 | 8.198 |
| D-MEM/F-12 media | 14.522 | 1.56 | 1.452 |
| Fetal Bovine Serum | 1.623 | 10 | 16.227 |
| Antibiotic/Antimycotic | 0.916 | 1 | 0.916 |
| NaHCO3 | 0.111 | 0.12 | 0.013 |
| 50 mL Tubes | 0.319 | 4 | 1.276 |
| 15 mL Tubes | 0.241 | 4 | 0.963 |
| MilliQ Water | 0.041 | 89 | 6.372 |
| Alcohol (70%) | 0.004 | 182.292 | 0.666 |
| Serological Pipettes (5mL) | 0.210 | 28 | 5.889 |
| Serological Pipettes (10mL) | 0.219 | 6 | 1.316 |
| Pipette Tips (10 uL) | 0.0116 | 8 | 0.093 |
| Tripan Blue | 0.0003 | 40 | 0.015 |
| Microtube (1.5 mL) | 0.018 | 4 | 0.070 |
| | | **SUM (USD $/batch)** | 55.150 |
| **ATPS Recovery** | | | |
| **Item** | **Cost per unit (USD $/g, USD$/mL, or USD $/piece)** | **Amount need for 90 mL of Cell culture (mL, g, or pieces)** | **Cost for 100 mL of Cell Culture** |
| PEG 10000 | 0.124 | 50.40 | 6.272 |
| Dextran 10000 | 3.914 | 135 | 529.049 |
| NaCl | 0.024 | 1314.9 | 31.605 |
| MilliQ Water | 0.041 | 346.50 | 25.397 |
| | | **SUM (USD $/batch)** | 592.32 |
| | | **TOTAL SUM (USD $/batch)** | 647.47 |
| | | **Exosome Protein per Batch (mg/batch)** | 161.70 |
| | | **Production Cost per mg of Exosome Protein (USD $/mg)** | 4.004 |
